# Transcriptomic analysis reveals the differentially expressed genes and pathways involved in drought tolerance in pearl millet [*Pennisetum glaucum* (L.) R. Br]

**DOI:** 10.1371/journal.pone.0195908

**Published:** 2018-04-13

**Authors:** Ambika Dudhate, Harshraj Shinde, Daisuke Tsugama, Shenkui Liu, Tetsuo Takano

**Affiliations:** 1 Asian Natural Environmental Science Center (ANESC), the University of Tokyo, Nishitokyo-shi, Tokyo, Japan; 2 Hokkaido University, Hokkaido, Japan; 3 State Key Laboratory of Subtropical Silviculture, Zhejiang A and F University, Lin’an, Hangzhou, China; Aberystwyth University, UNITED KINGDOM

## Abstract

Pearl millet is a cereal crop known for its high tolerance to drought, heat and salinity stresses as well as for its nutritional quality. The molecular mechanism of drought tolerance in pearl millet is unknown. Here we attempted to unravel the molecular basis of drought tolerance in two pearl millet inbred lines, ICMB 843 and ICMB 863 using RNA sequencing. Under greenhouse condition, ICMB 843 was found to be more tolerant to drought than ICMB 863. We sequenced the root transcriptome from both lines under control and drought conditions using an Illumina Hi-Seq platform, generating 139.1 million reads. Mapping of sequenced reads against the foxtail millet genome, which has been relatively well-annotated, led to the identification of several differentially expressed genes under drought stress. Total of 6799 and 1253 differentially expressed genes were found in ICMB 843 and ICMB 863, respectively. Pathway and gene function analysis by KEGG online tool revealed that the drought response in pearl millet is mainly regulated by pathways related to photosynthesis, plant hormone signal transduction and mitogen-activated protein kinase signaling. The changes in expression of drought-responsive genes determined by RNA sequencing were confirmed by reverse-transcription PCR for 7 genes. These results are a first step to understanding the molecular mechanisms of drought tolerance in pearl millet and lay a foundation for its genetic improvement.

## Introduction

Drought is a major abiotic stress that adversely affects agricultural productivity worldwide. Most of the cereal crops on which the world population depends for food are susceptible to drought. Water deficiency is a global concern that is expected to become worse in the next decades [1]. Therefore, understanding the mechanisms of drought tolerance in cereal crops and development of drought-tolerant varieties are key strategies to maintain yield under drought conditions. Mechanisms that overcome drought stress in plants include drought avoidance, drought tolerance, drought escape and drought recovery [2,3]. At the molecular level, plants respond to drought by inducing both regulatory and functional genes [4]. Many metabolic pathways, such as the abscisic acid (ABA) signaling pathway, mitogen-activated protein kinase (MAPK) signaling pathway and photosynthesis-associated pathways, respond to drought stress [5–7]. Recently many efforts have been made to elucidate the mechanism of drought tolerance in non-model cereal crops through molecular and genomic approaches [8–10].

Pearl millet (*Pennisetum glaucum* (L.) R. Br.) (2n = 2X = 14) is the world’s sixth most important cereal crop, and is primarily grown as a rain-fed crop in the low rainfall zones of Sub-Saharan Africa and the Indian subcontinent [11]. Pearl millet is a C4 cereal crop belonging to the family Poaceae and subfamily Panicoideae. It is a cross-pollinated crop with short life cycle and outbreeding nature. [12]. Compared to other cereal crops such as rice, wheat, maize and sorghum, it has high tolerance to abiotic stresses, such as drought, salinity, high temperature and soil nutrient deficiency [13]. It is mainly grown for food grain production, but in some cases, it is also grown for hay, bird feed, biofuel and forage. Pearl millet is a nutrient-rich crop; its seeds contain more protein, iron, zinc and energy than seeds of rice, wheat and maize [14].

So far only two research groups have investigated drought tolerance in pearl millet using transcriptomic approaches [15,16]. A few genes, such as *PgDREB2A* (Drought responsive element binding protein 2A) and *PgGPx* (Glutathione peroxide), have been studied for their role in drought tolerance [17,18]. However, it is expected that many more genes are involved. Recently, the pearl millet genome has been sequenced by the international pearl millet genome sequencing consortium (IPMGSC). The draft genome sequence is publicly available, and will help to understand the pearl millet drought tolerance mechanisms [19], although it needs to be further annotated.

Transcriptomic analysis using next generation sequencing (NGS) is an efficient method for exploring gene expression patterns in plants. However, RNA Sequencing (RNA-Seq) using NGS has the ability to detect differentially expressed genes (DEGs) because it has a dynamic range of expression levels [20]. Here, we used an RNA-Seq approach to understand the pathways involved in pearl millet in responses to drought stress. The data will help to design more detailed studies of drought tolerance in this species as well as develop DNA markers that could facilitate breeding more drought-tolerant varieties.

## Materials and methods

### Experimental material and growth condition

Seeds of pearl millet were procured from International Crop Research Institute of Semi-arid Tropics (ICRISAT), India. Two inbred lines ICMB 843 as drought tolerant and ICMB 863 as comparatively less drought tolerant having different levels of drought tolerance were selected for our study.

The experiment was conducted under controlled greenhouse conditions. Around 20 seeds of each inbred line were sown in equal volumes of soil and vermiculite in perforated terracotta pots with the size of 11 litters. Each growth experiment was done with three replications. For growth tests and RNA-Seq, drought stress was imposed on 21-day-old plants by withholding water for 5 days, while control plants were watered on alternate days.

Plants of both lines were grown as described in the “Experimental material and growth condition” subsection. SPAD values of control and drought stressed plants were measured using SPAD 502 Plus Chlorophyll Meter (Spectrum Technologies Inc., Aurora, Illinois, USA). Relative water content (RWC) was calculated as previously described [21].

### RNA extraction, library preparation and sequencing

Roots of drought-stressed plants and control plants were ground in liquid nitrogen with a mortar and pestle. Total RNA was isolated with Trizol (Sigma-Aldrich Cat.no:15596026), and genomic DNA was digested with RNase-Free DNase (Qiagen). RNA degradation and potential DNA contamination were checked by gel electrophoresis. RNA integrity number (RIN) and quantitation check were performed by Agilent 2100 bioanalyzer. After the quality check procedures, mRNA from roots was enriched using oligo(dT) beads. The mRNA was fragmented randomly by adding fragmentation buffer, then the cDNA was synthesized using mRNA template and random hexamers, after which a second strand synthesis buffer (Illumina), dNTPs, RNase H and DNA polymerase I were added to initiate the second-strand synthesis. After terminal repair and sequencing adapter ligation, the double-stranded cDNA library was completed through size selection and PCR enrichment. The qualified libraries were subjected to sequencing by Illumina Hiseq through NOVOgene Co., ltd. Twelve data sets for paired-end sequencing reads in FASTQ format were submitted to SRA (Sequence Read Archive) at NCBI, and can be retrieved by the accession number SRP125789.

### Reads mapping and differential gene expression analysis

Raw reads obtained by sequencing were quality-checked using online tool FASTQC (Version.0.11.5). Qualified reads were mapped to the reference genomes using Genome Work bench 9.5.4 (CLC Bio. Japan). The read counts were normalized by reads per kilobase per million mapped reads (RPKM = Total exon reads/mapped reads in million X exon length in kb) for each gene and log_2_ transformed.

Gene expression levels were estimated by RPKM values using advanced plugins for RNA-Seq in CLC Workbench 9.5.4. Baggereley test was used to calculate *P* values. Genes with log_2_ fold changes >2 or < -2, and FDR-corrected p values <0.05 were regarded as DEGs.

### Pathway enrichment analysis

The DEGs in both inbred lines of pearl millet (ICMB 843 and ICMB 863) under control and stressed conditions were mapped to the biological pathways by using online tool KEGG (Kyoto encyclopedia for genes and genomes) [22] and the pathways enriched by DEGs were studied further.

### Validation of RNA-Seq data by real time RT-PCR

Seven genes were selected based on the RPKM values. Real time PCR was performed using Step-one Real-Time instrument (Applied Biosystems) and the SYBR Green I kit (Roche, Basel, Switzerland). Reactions were performed in triplicate and contained 100 ng of cDNA, 1 μL of each primer (10 μM/ μL) and 10 μL of SYBR green master mix in a final volume of 20 μL. The primer pairs listed in S1 Table were designed to yield products with the sizes of 80–200 bp based on the sequences in the foxtail millet genome mapped with the pearl millet reads. The PCR cycling conditions were as follows: an initial denaturation step of 20 s at 50 °C, 10 min at 95 °C, and 40 cycles of 15 s at 95 °C, and 1 min at 60 °C followed by melt curve analysis. The *PgActin* gene was used as a reference gene [23]. Relative fold differences for each sample in each experiment were calculated using the ΔΔCt method [24].

## Results

### Effect of drought treatment on physiology of pearl millet lines

**Fig 1:**
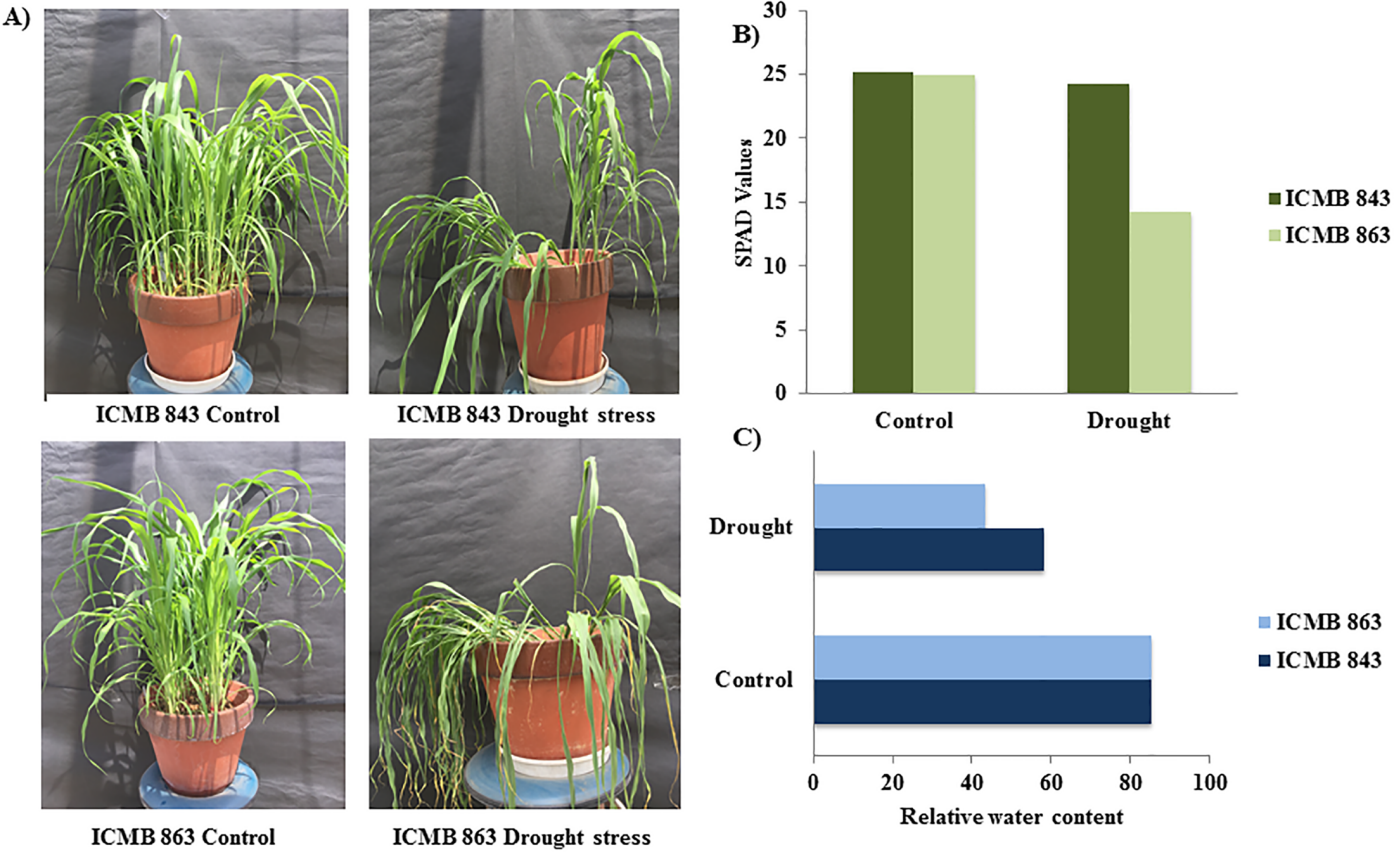
Physiological analysis of pearl millet for drought. A) Effects of drought on the physiology of pearl millet lines (ICMB 843 and ICMB 863). B) SPAD value of pearl millet lines under and drought conditions. Values are mean ± SE (n = 20) C) Relative water content of leaf under control and drought stress. Values are mean ± SE (n = 20).

Within 5–7 days of drought stress treatment, ICMB 863 started showing drought stress symptoms such as leaf tip drying and rolling, whereas ICMB 843 did not show any of these symptoms. Both lines showed lodging during drought stress, but lodging was more severe in ICMB 863 (Fig 1A). Total chlorophyll content or greenness determined by SPAD meter (Fig 1B) showed a greater reduction of chlorophyll in ICMB 863 than in ICMB 843. These observations indicate that the drought caused a greater reduction in photosynthetic activity in ICMB 863 than in ICMB 843. Drought treatment decreased RWC significantly more in ICMB 863 than in ICMB 843 (Fig 1C). These results suggest that ICMB 843 is more tolerant to drought than ICMB 863 at seedling stage.

### Transcriptomic profiling and mapping statistics of pearl millet

Roots are the organ that would first respond to drought stress, and drought-stressed pearl millet roots were subjected to RNA-Seq to analyze their transcriptome. Illumina sequencing resulted in generating around 139 million of raw reads. Preprocessing with the CLC genomics workbench 9.0 generated 134 million clean reads (Table 1).

**Table 1:** Basic statistics for sequenced reads from transcriptome of pearl millet.

Samples	Raw reads	Clean reads	Q20(%)**	Q30(%)***	Total mapped****
ICMB843_control^1^	11615061	11188437	93.17	84.48	14,208,882(63.48%)
ICMB843_control^2^	11934447	11340253	93.58	85.21	13,546,964(59.73%)
ICMB843_control^3^	11469576	11070226	93.44	84.88	12,913,182(58.32%)
ICMB843_Treated^1^	7648776	7433785	93.66	85.77	5,482,220(36.87%)
ICMB843_Treated^2^	13014255	12566731	94.78	87.97	6,049,930(24.07%)
ICMB843_Treated^3^	11678697	11382308	93.61	85.69	8,638,226(37.95%)
ICMB863_control^1^	10735082	10169174	93.65	85.86	110,10,118(54.17%)
ICMB863_control^2^	10029364	9297019	93.97	85.30	8,582,854(46.16%)
ICMB863_control^3^	14717884	14570208	95.05	85.86	17,240,062(59.16%)
ICMB863_Treated^1^	10551898	10249771	94.33	88.84	10,256,990(50.04%)
ICMB863_Treated^2^	16115349	15657629	94.47	87.65	19,234,298(61.42%)
ICMB863_Treated^3^	9614712	9342152	93.41	85.33	10,034,136(53.70%)
Total	139125101	134267693			126,187.774

^1,2,3^ = Individual Biological replications of sample

**Q20(%) = Bases with the phred value >20 (in percentage)

***Q30(%) = Bases with the phred value >30 (in percentage)

****Total number of reads mapped to the genome of *Setaria Italica* (percentage)

**Fig 2:**
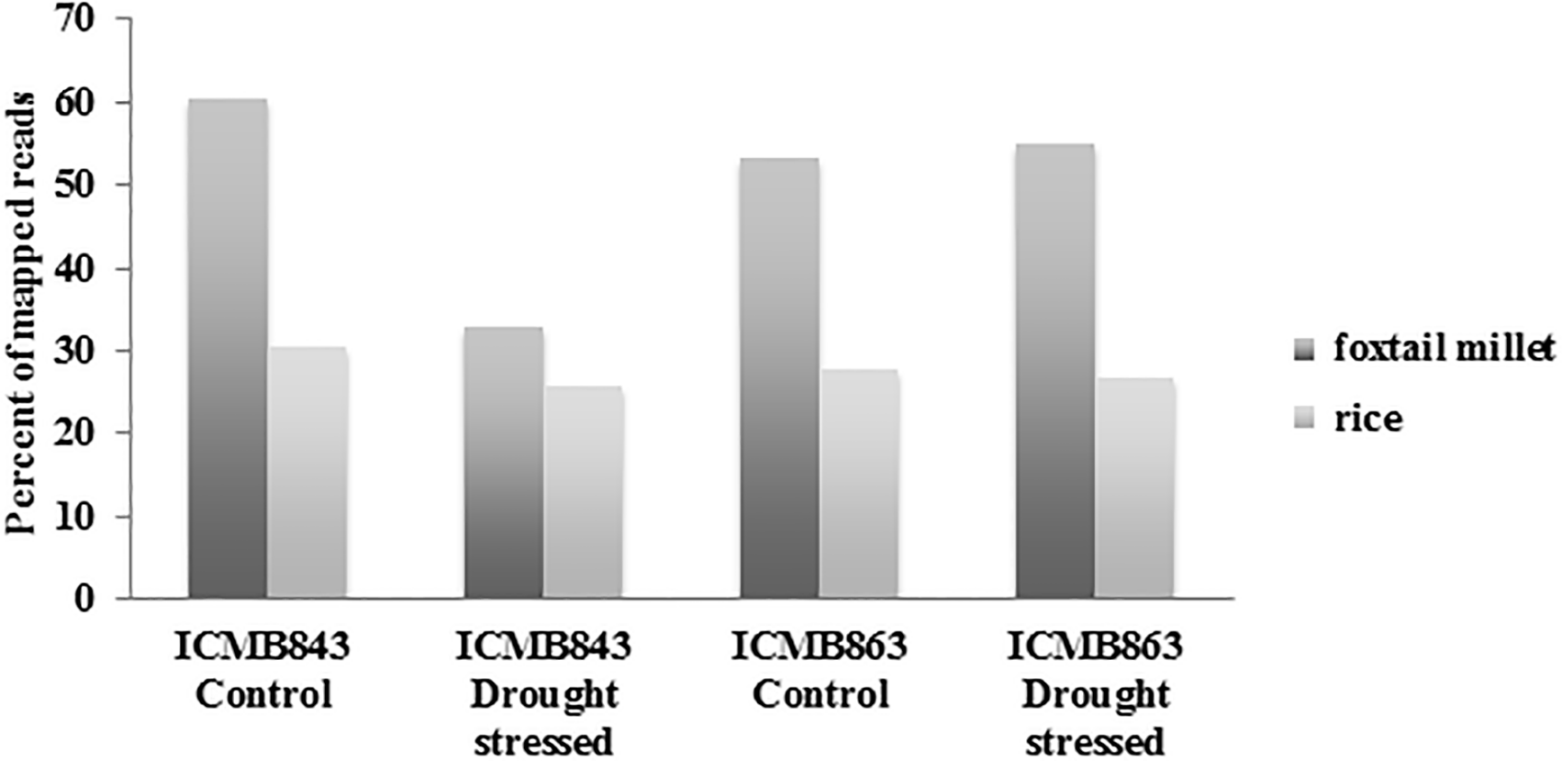
Mapping of pearl millet reads to the foxtail millet genome and the rice genome. X axis denotes the sample names under control and drought stress and Y axis denotes the percentage of mapped genes.

The clean reads were mapped to the genomes of two closely related members of the Poaceae family, foxtail millet (*S*. *italica*) and rice (*O*. *sativa*) [25]. These genomes were used because they have been annotated better than the pearl millet genome. About 33–61% of the reads were mapped to the foxtail millet genome, and about 25–30% of the reads were mapped to the rice genome (Fig 2). Therefore, the foxtail millet genome was used as the reference genome for further analyses. Interestingly, the percentage of mapped reads was lower in the samples from drought-stressed ICMB 843 than in the other samples (Fig 2 and Table 1). This might be because drought stress increases variation in transcripts in ICMB 843.

### Differentially expressed genes for drought stress in pearl millet

**Fig 3:**
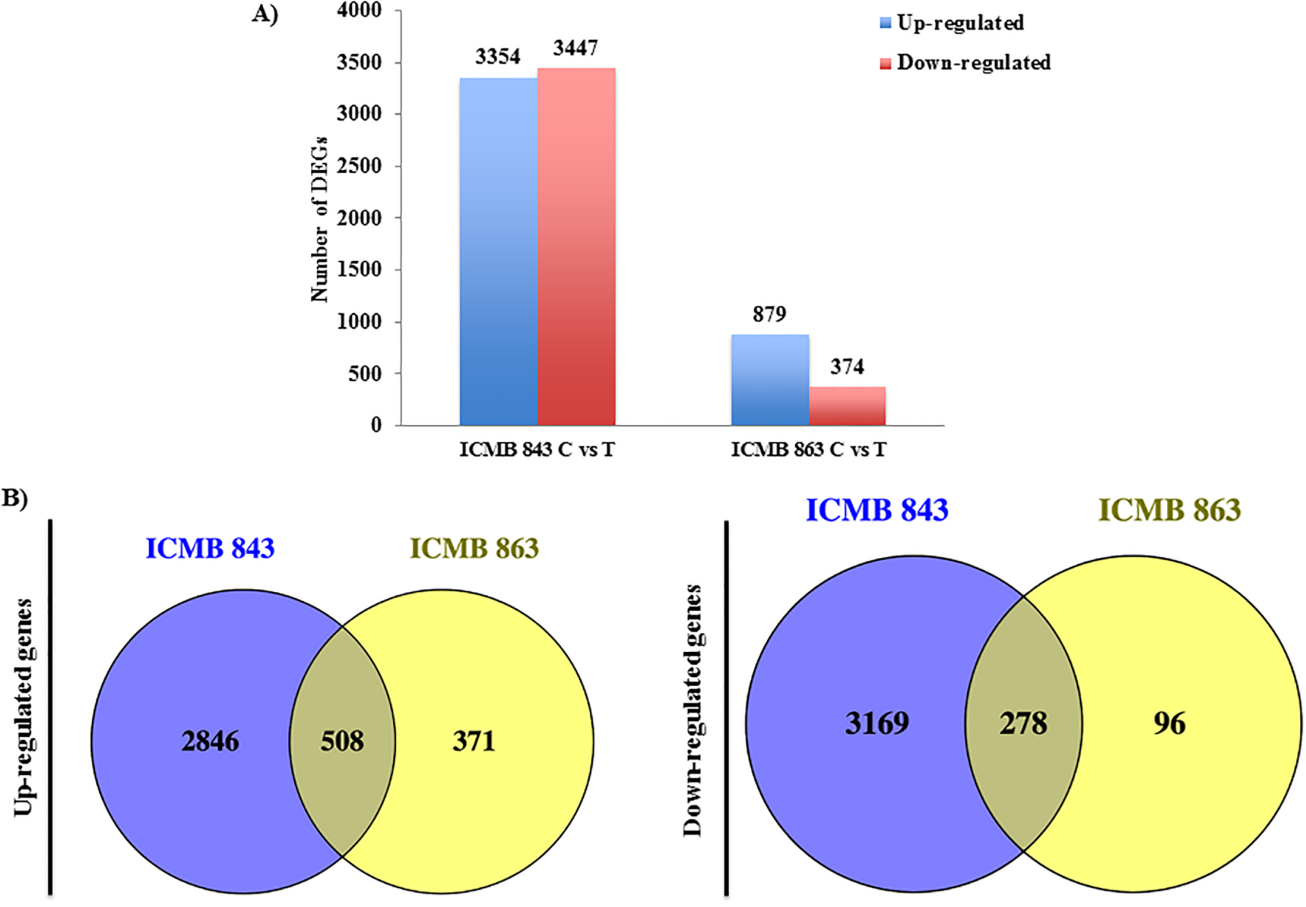
DEG analysis in comparisons study. A) Number of DEGs in all combinations with fold change >2 or <-2 and FDR-corrected *p*value <0.05) Blue and red bars indicate up- and down- regulated respectively. C: control and T and Treated: Drought- treated B) Venn diagram showing an up-regulated (right side) and down regulated (left side) genes in lines ICMB 843 and ICMB 863 under drought treated situation.

RPKM and log_2_ transformation were used for extracting DEGs. Drought treatment up-regulated ~4 times more genes in the drought-tolerant line ICMB 843 than in the drought-sensitive line ICMB 863, and while it down-regulated ~10 times more genes in ICMB 843 than in ICMB 863 (Fig 3A).

Venn diagram highlights overlapping DEGs expressed in both ICMB 843 and ICMB 863 under drought situation (Fig 3B). In ICMB 843, 2846 genes were up-regulated and 3169 were down-regulated by drought treatment, while in ICMB 863 about 371 genes were up-regulated and 96 genes were down-regulated. Common genes that were up-regulated and down-regulated in both lines were 508 and 278 respectively.

### Effect of drought on biological pathways of pearl millet

**Fig 4:**
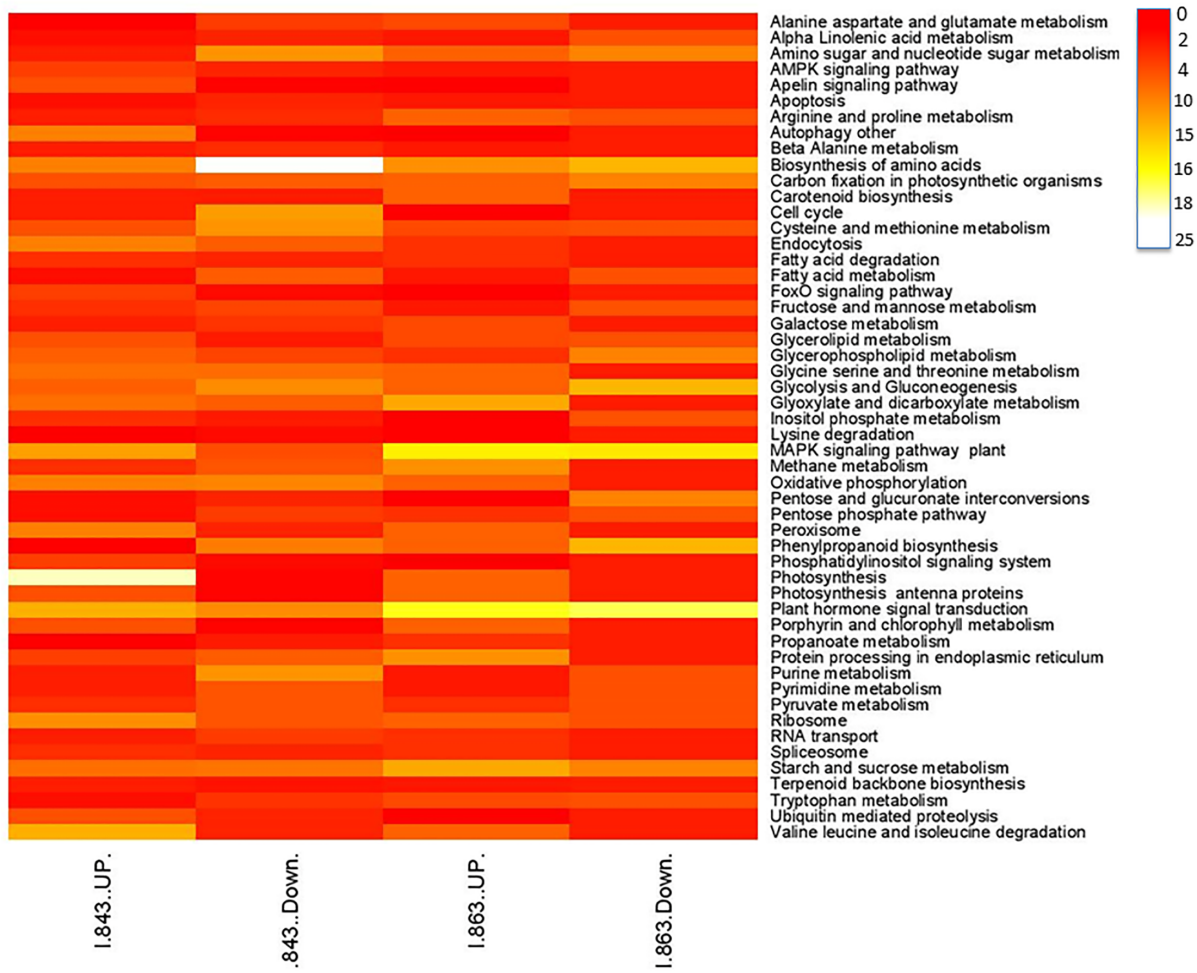
Heat map showing the number of DEGs enriched in different KEGG pathways. The bar scale shows the number of DEGs associated with pathways.

To associate the DEGs found in our RNA-Seq with known biological pathways, KEGG analysis was performed. The biological pathways enriched with up-regulated DEGs were photosynthesis, plant hormone signal transduction and MAPK signal transduction (Fig 4).

**Fig 5:**
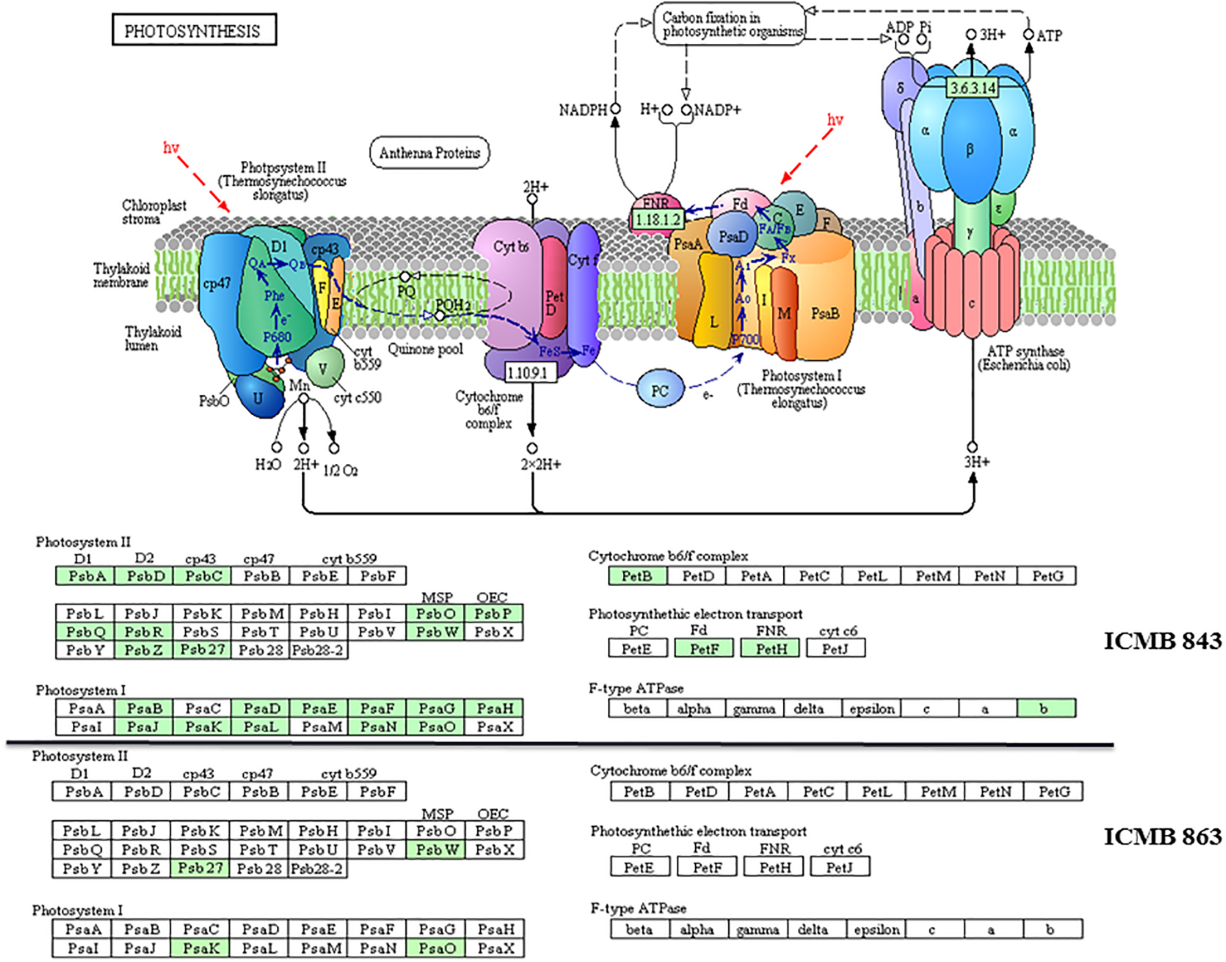
DEGs involved in photosynthesis. Genes up-regulated by drought stress are shown (25 genes in ICMB 843 and 4 in ICMB 863) in green boxes. White boxes indicate the non-drought-responsive genes. This image was generated using the online tool KEGG Mapper–Colour Pathway (http://www.genome.jp/kegg/tool/map_pathway3.html).

There were around 25 upregulated genes encoding components of five subunits of photosynthesis (Photosystem II, Photosystem I, cytochrome b6/f complex, photosynthetic electron transport and f-type ATPase). Most of them were up regulated in ICMB 843 but not in ICMB 863 (Fig 5).

**Fig 6:**
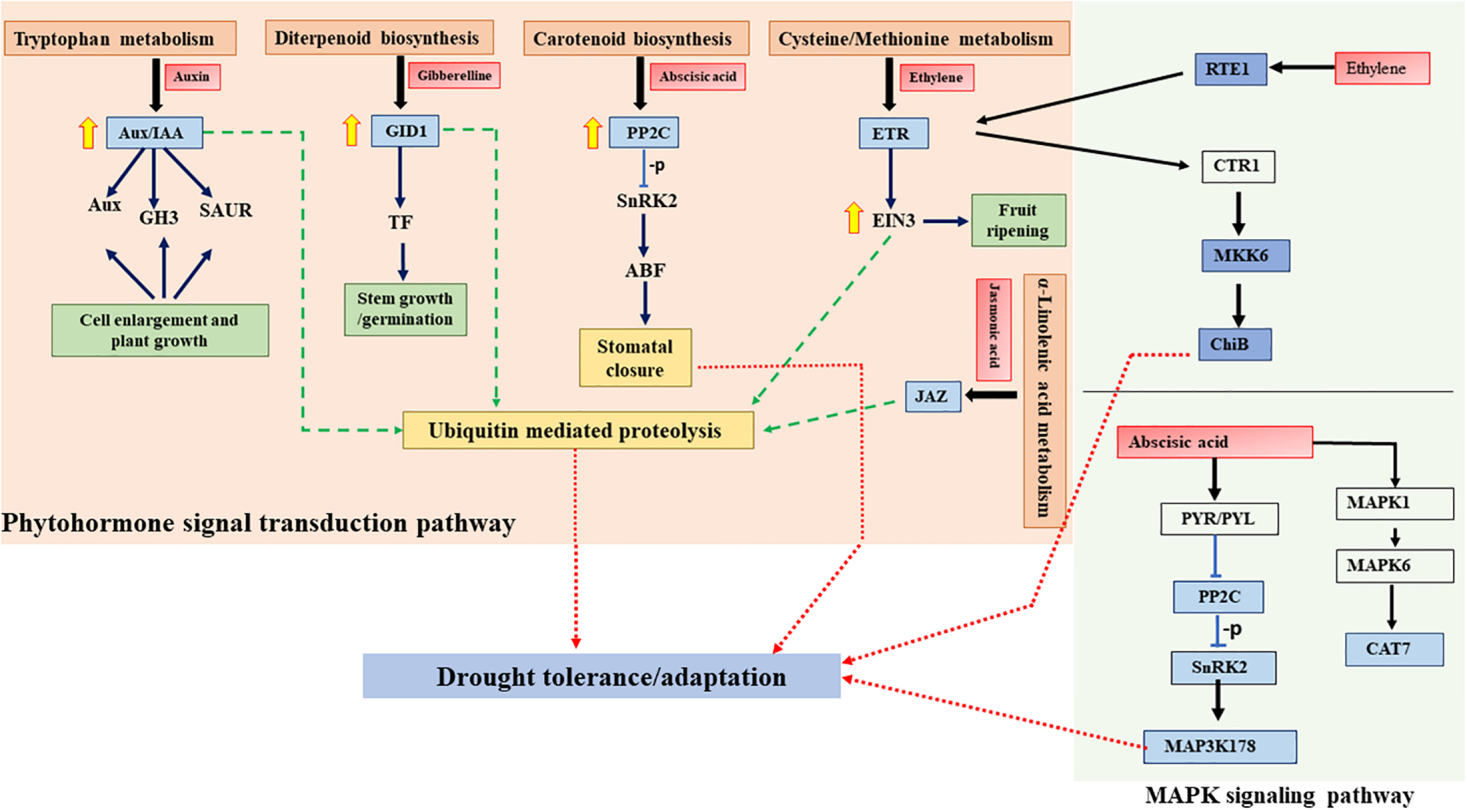
DEGs involved in phytohormone and MAPK signaling pathway. The proposed pathways show phytohormone biosynthesis (light orange) and MAPK signaling pathways (light blue). This figure shows only the genes that have been associated with the pathway from transcriptome analysis. Red boxes show plant hormones, blue boxes show the genes up-regulated stress, green arrow shows genes involved in drought tolerance through ubiquitin mediated proteolysis and red arrows shows the direct involvement in the drought tolerance. Light blue lines with a bar denotes inhibition and simple arrows denotes activation. -p indicates de phosphorylation process. Aux/IAA: auxin-responsive protein IAA; GH3: Auxin-responsive GH3 family protein; SAUR: SAUR-like auxin-responsive protein family; CRE: Histidine kinase 2/3/4 (cytokinin receptor); B-ARR: Two-component response regulator ARR-B family; GID1: Alpha/beta-Hydrolases superfamily protein; TF: Phytochrome interacting factor 3 (PIF3); PP2C: Protein phosphatase 2C; SnRK2: Serine/threonine-protein kinase SRK2; ABF: ABA responsive element binding factor; ETR: Ethylene receptor; EIN3: ETHYLENE-INSENSITIVE3-like 3; JAZ: Jasmonate ZIM domain-containing protein; RTE1: Transmembrane protein 222; MKK9: Mitogen-activated protein kinase kinase 9; ChiB: Basic endochitinase B; MAP3K17/18: Mitogen-activated protein kinase kinase kinase 17/18; MAPK1: Mitogen-activated protein kinase 1; MAPK6: Mitogen-activated protein kinase 6; CAT7: Catalase.

In total, 22 genes in our study shows association with these two signaling pathways. These 22 genes were mainly related to the genes with Auxin biosynthesis (*Aux/IAA*, *GH3*, *SAUR*), Diterpenoid biosynthesis (*GID1*, *PIF*), Carotenoid biosynthesis (*PP2C*, *SnRK2*, *ABF*), Ethylene biosynthesis (*ETR*, *RTE*, *MKK6*, *ChiB*) and Abscisic acids (*PVR*, *MAP3K17/18*, *CAT1*, *MAPK1*, *MAPK6*). Further details about the pathways and associated genes are listed in the (S2 Table). Plant hormone signaling pathway and MAPK pathways seems to be interlinked with each other as they share common byproducts that may increase tolerance to drought in pearl millet (Fig 6).

### Validation of RNA-Seq data with the quantitative real time PCR

**Fig 7:**
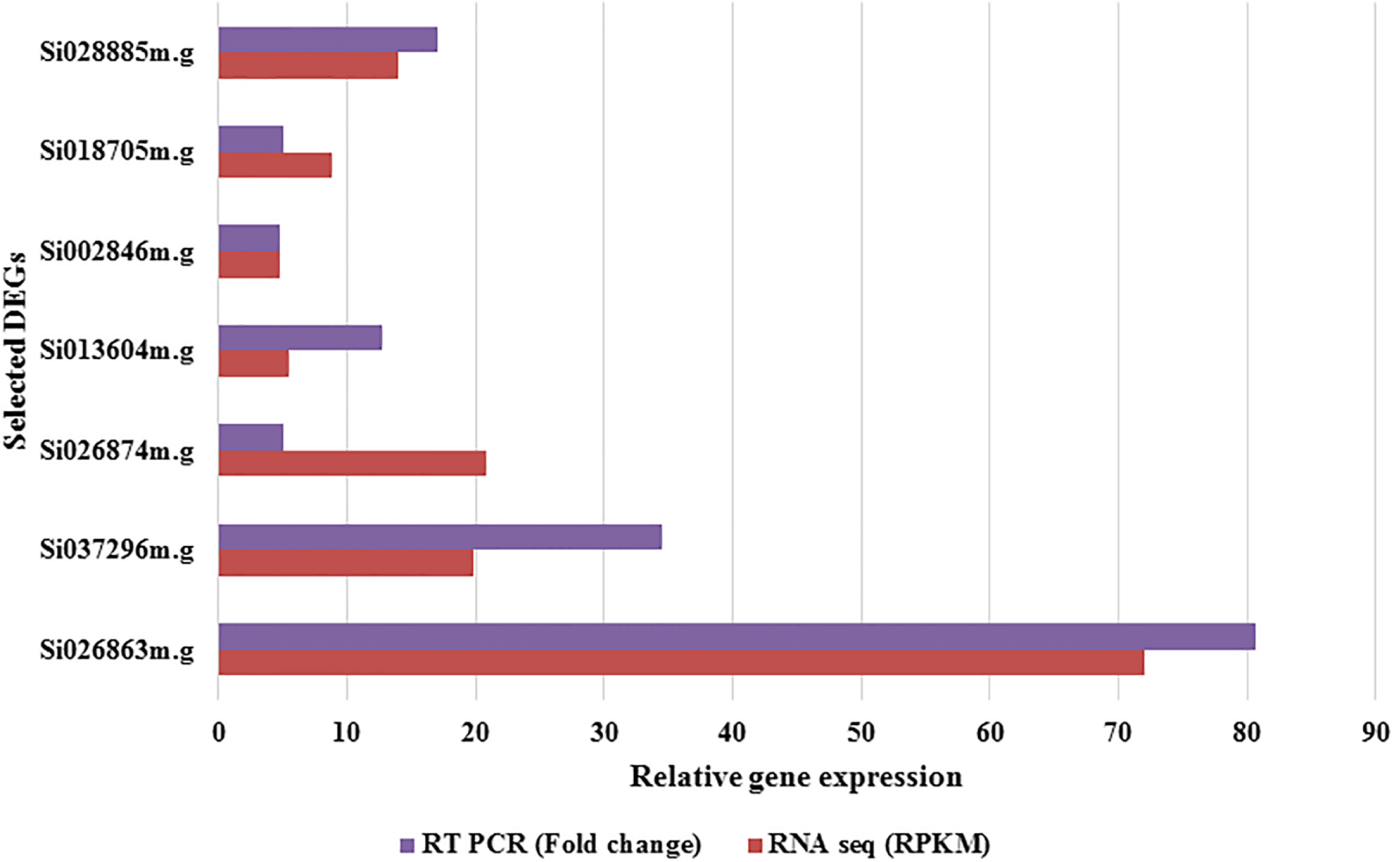
Validation of RNA-Seq result with RT PCR. Expression of 7 randomly selected genes was examined by RT- PCR analysis. For each gene, fold changes were calculated by ΔΔCt method in the RT-PCR and with the RPKM values in the RNA-Seq, respectively.

The relative gene expression patterns of the qRT-PCR results for 7 genes were consistent with RNA-Seq data (Fig 7). The annotations for the genes selected for validation are listed in (Table 2).

**Table 2:** Annotations of the genes selected for RT-PCR.

Name of the gene (Identifier)	Annotations	Fold (RT PCR)
Si028885m.g	Mixed linked glucan synthase	1.9
Si018705m.g	Chlorophyll centre-I related protein	5.11
Si002846m.g	Dehydration responsive elements binding protein 1F	4.45
Si013604m.g	Uncharacterized protein	12.8
Si026874m.g	Uncharacterized protein	5.1
Si037296m.g	Glutathione s-transferase (GSTU6)	34.5
Si026863m.g	Abiotic stress responsive factor	80.7

## Discussion

Many drought-responsive genes were identified by our RNA-Seq analysis. Many of these genes were associated with photosynthesis, plant hormone signaling and MAPK signaling pathway. To our knowledge, this is the first study in pearl millet for drought tolerance by RNA-Seq approach.

### Drought responsive genes involved in photosynthesis

As a primary metabolic process, photosynthesis plays a crucial central role in plants life when under drought stress. One of the major effects of drought and other environmental stresses [7,26] is to damage both of the Photosystems (PS I and PS II, respectively) [27]. PS II is a complex network of protein containing several types of chlorophyll binding components. Main function of these components is to organize chlorophyll for light harvesting but PS II also acts as cofactor needed for oxidation of water [28]. In our study, we found that 25 up-regulated genes in ICMB 843 and 8 genes in ICMB 863 are involved in photosynthesis and photosynthesis-related pathways. In Arabidopsis, PS II and light harvesting complex II (LHC II) play an important role in preventing the photo-damage to PS II under drought stress [29]. Ten PS II genes were up-regulated in ICMB 843 upon drought stress. These included *PsbQ*, which is necessary for regulation of activity of PS II [30], *PsbR*, which is required for preparation of oxygen evolving complex of PS II, *PsbP*, *PsbQ* and *PsbW*, which stabilizes the supramolecular organization of PS II and six other genes (*PsbA*, *PsbD*, *PsbC*, *Psb I*, *PsbZ*, *Psb27*) [31]. On the other hand, in ICMB 863, only *PsbW* and *Psb27* genes were up-regulated. As mainly these genes are related to avoid photodamage to PS II, it might be possible that on drought stress pearl millet activates the genes to maintain its structural integrity. Other up-regulated genes such as *PsaB PsaD* and *PsaE* are related to electron transport and ferredoxin binding [32]. PsaF, PsaG, PsaH, PsaK, PsaL, PsaK and PsaO have a role in binding of light harvesting complex [33] and genes encoding them were also found to be up-regulated by drought stress (Fig 5 and S2 Table). These results raise the possibility that electron transport in PS I and PS II is activated by drought stress in pearl millet.

### Drought and plant hormone signal transduction and MAPK signalling

Phytohormones, such as abscisic acid (ABA), cytokinin (CK), gibberellic acid (GA), auxin, and ethylene, and jasmonate regulate diverse processes and confers drought tolerance to plants [34]. In our analysis, we found genes associated with the biosynthesis and signalling of these phytohormones. Such genes include *AUX/IAA*, *GH3*, and *SAUR*. Auxin biosynthesis is known to confer drought tolerance in Rice [35], *OsGH3-2* a member of the *GH3* family was proved to be modulating the level of ABA and stress tolerance in rice [36], *SAUR* genes has not been yet functionally characterized, are altered by auxin and are hypothesized to be involved in the drought tolerance by auxin [37]. *GID1* and *Phytochrome interacting factor 4* are involved GA signaling and in regulating drought stress responses [38,39]. *PP2C*, *SnRK2*, and *ABRF* are involved in ABA signaling and in regulating drought stress [40–42]. Our study suggests that homologs of these genes are all up-regulated by drought stress in pearl millet. Biosynthesis of ethylene from 1-aminocyclopropane-1-carboxylic acid during drought is triggered to induce the senescence [43]. We found up- regulation of pearl millet homologs encoding *ETR* and *EIN3* which were previously showed to mediate ethylene signaling and to negatively regulate drought stress [44–46]. Further studies are needed to elucidate their relevance to drought stress response of pearl millet. Biosynthesis, modification, and signaling of jasmonate during drought are comprehensively studied [47]. Up-regulation of homologs possibly encoding *JAZ* protein which mediates jasmonate signaling was observed in our study.

MAPK is a signal transduction pathway somewhat conserved in all eukaryotes, including yeasts, animals and plants; involvement of MAPK in drought stress responses has been already reported [48]. RNA-Seq analysis of maize found that MAPK kinase kinases (MAPKKKs) have important regulatory functions in drought tolerance in maize [49]. In our study of pearl millet, we observed the drought-responsive up-regulation of MAPK signaling pathway. Some other components of MAPK cascades are known to be involved in ABA and ethylene signaling and drought stress responses. The Upregulation of these kinases may contribute to conferring drought tolerance in case of the pearl millet (Fig 6 and S2 Table).

## Conclusions

Pearl millet is a drought-tolerant cereal crop and therefore represents a novel source for investigating the molecular mechanism of drought tolerance in cereal crops. Two pearl millet inbred lines, ICMB 843 and ICMB 863, which exhibit diverse responses against drought stress were used for the study. ICMB 843 is relatively more tolerant to drought than ICMB 863 at seedling stage. RNA-Seq analysis showed that ICMB 843 activated more number of drought responsive genes than ICMB 863. Genes related to photosynthesis, plant hormone signal transduction and MAPK signalling pathways were induced by drought in pearl millet. Identified drought- responsive genes and metabolic pathways are targets for future studies in order to understand the molecular mechanism of drought tolerance in pearl millet.

## Supporting information

S1 Table Primers used in RT PCR for validation of RNA seq data. (XLSX)

S2 Table Drought responsive expression pattern of selected DEGs in genotypes of pearl millet. (DOCX)
