# Supplementary material for: Transcriptomic analysis reveals the differentially expressed genes and pathways involved in drought tolerance in pearl millet [Pennisetum glaucum (L.) R. Br]
Source: PLoS One. 2018 Apr 13;13(4):e0195908. doi: 10.1371/journal.pone.0195908 (PMC5898751; doi:10.1371/journal.pone.0195908)
Supplement: S2 Table — (DOCX) [file pone.0195908.s002.docx]

**S2 Table : Drought responsive expression pattern of selected DEGs in genotypes of pearl millet**

| **Gene Id (mapped)** | **Annotations** | **ICMB 843 ICMB 863** | | | | | | | | | | | | | | | | |  |
| --- | --- | --- | --- | --- | --- | --- | --- | --- | --- | --- | --- | --- | --- | --- | --- | --- | --- | --- | --- |
|  |  | **FDR Log2FC Regulation** | | | | | | | | **FDR Log2FC Regulation** | | | | | | | | |  |
| Photosynthesis | | | | | | | | | | | | | | | | | | |  |
| Si012451 | Photosystem II P680 Reaction Center D1 Protein | 2.84E-15 | | 8.07 | UP | | | | | | - | | | | - | | - | |  |
| Si008469 | Photosystem II P680 Reaction Center D2 Protein | 1.43E-03 | | 4.31 | UP | | | | | | - | | | | - | | - | |  |
| Si005073 | Photosystem II CP43 Chlorophyll Apoprotein | 7.88E-05 | | 6.09 | UP | | | | | | - | | | | - | | - | |  |
| Si002174 | Photosystem II Oxygen-Evolving Enhancer Protein 1 | 0 | | 4.64 | UP | | | | | | - | | | | - | | - | |  |
| Si003335 | Photosystem II Psbw Protein | 0 | | 5.67 | UP | | | | | | 0.0307 | | | | 2.40 | | UP | |  |
| Si0037195 | Photosystem II Oxygen-Evolving Enhancer Protein 2 | 4.45E-07 | | 1.96 | UP | | | | | | - | | | | - | | - | |  |
| Si031101 | Photosystem II Oxygen-Evolving Enhancer Protein 3 | 0 | | 3.58 | UP | | | | | | - | | | | - | | - | |  |
| Si014601 | Photosystem II 10kda Protein | 9.87E-13 | | 7.84 | UP | | | | | | - | | | | - | | - | |  |
| Si039577 | Photosystem II Psbz Protein | 0.03426 | | 5.91 | UP | | | | | | - | | | | - | | - | |  |
| Si038607 | Photosystem II Psb27 Protein | 0 | | 4.08 | UP | | | | | | 0.0239 | | | | 2.20 | | UP | |  |
| Si037249 | Photosystem I P700 Chlorophyll A Apoprotein A2 | 3.02E-03 | | 5.22 | UP | | | | | | - | | | | - | | - | |  |
| Si037566 | Photosystem I Subunit II | 8.31E-15 | | 4.33 | UP | | | | | | - | | | | - | | - | |  |
| Si031265 | Photosystem I Subunit IV | 9.13E-14 | | 6.93 | UP | | | | | | - | | | | - | | - | |  |
| Si037323 | Photosystem I Subunit III | 8.62E-04 | | 3.26 | UP | | | | | | - | | | | - | | - | |  |
| Si031456 | Photosystem I Subunit V | 6.04E-05 | | 5.30 | UP | | | | | | - | | | | - | | - | |  |
| Si023570 | Photosystem I Subunit VI | 0 | | 4.44 | UP | | | | | | - | | | | - | | - | |  |
| Si014853 | Photosystem I Subunit IX | 0.01751 | | 6.61 | UP | | | | | | - | | | | - | | - | |  |
| Si031478 | Photosystem I Subunit X | 0 | | 3.86 | UP | | | | | | 0.0116 | | | | 2.35 | | UP | |  |
| Si023254 | Photosystem I Subunit XI | 0 | | 5.67 | UP | | | | | | - | | | | - | | - | |  |
| Si011251 | Photosystem I Subunit Psan | 5.30E-03 | | 7.64 | UP | | | | | | - | | | | - | | - | |  |
| Si011260 | Photosystem I Subunit Psao | 3.99E-07 | | 7.71 | UP | | | | | | 2.34E-03 | | | | 3.60 | | UP | |  |
| Si024707 | Cytochrome B6 | 0.01960 | | 3.87 | UP | | | | | | - | | | | - | | - | |  |
| Si003262 | Ferredoxin | 0.02685 | | 3.38 | UP | | | | | | - | | | | - | | - | |  |
| Si017591 | Ferredoxin--NADP+ Reductase | 1.07E-08 | | 4.56 | UP | | | | | | - | | | | - | | - | |  |
|  |  |  | |  |  | | | | | |  |  | | | | |  | |  |
| Plant Hormone Signal Transduction | | | | | | | | | | | | | | | | | | |  |
| Tryptophan Metabolism | | | | | | | | | | | | | | | | | | |  |
| Si022572 | Auxin-Responsive Protein IAA | 1.41E-06 | 1.23 | | | UP | | | - | | | | - | | | - | | |  |
| Si039741 | Auxin Responsive GH3 Gene Family | 0.03375 | 3.42 | | | UP | | | - | | | | - | | | - | | |  |
| Si018749 | SAUR Family Protein | 0 | 1.89 | | | UP | | | - | | | | - | | | - | | |  |
| Zeatin Biosynthesis | | | | | | | | | | | | | | | | | | |  |
| Si000168 | Arabidopsis Histidine Kinase 2/3/4 (Cytokinin Receptor) | 9.93E-09 | 1.19 | | | UP | | | - | | | | - | | | - | | |  |
| Si016630 | Two-Component Response Regulator ARR-B Family | 1.85E-09 | 1.25 | | | UP | | | - | | | | - | | | - | | |  |
|  |  |  |  | | |  | | |  | | | |  | | |  | | |  |
| Diterpenoid Biosynthesis | | | | | | | | | | | | | | | | | | |  |
| Si022513 | Gibberellin Receptor GID1 | 2.62E-08 | 1.15 | | | UP | | | - | | | | - | | | - | | |  |
| Si022196 | Phytochrome-Interacting Factor 4 | 7.73E-08 | 3.83 | | | UP | | | - | | | | - | | | - | | |  |
|  |  |  |  | | |  | | |  | | | |  | | |  | | |  |
| Carotenoid Biosynthesis | | | | | | | | | | | | | | | | | | |  |
| Si001708 | Protein Phosphatase 2C | 0 | 1.89 | | | UP | | | - | | | | - | | | - | | |  |
| Si036041 | Serine/Threonine-Protein Kinase SRK2 | 0 | 2.12 | | | UP | | | - | | | | - | | | - | | |  |
| Si017618 | ABA Responsive Element Binding Factor | 7.51E-12 | 1.58 | | | UP | | | - | | | | - | | | - | | |  |
|  |  |  |  | | |  | | |  | | | |  | | |  | | |  |
| Cystein And Methionine Metabolism | | | | | | | | | | | | | | | | | | |  |
| Si016376 | Ethylene Receptor | 0 | 4.43 | | | UP | | | - | | | | - | | | - | | |  |
| Si009909 | Ethylene-Insensitive Protein 3 | 2.11E-03 | 4.92 | | | UP | | | - | | | | - | | | - | | |  |
|  |  |  |  | | |  | | |  | | | |  | | |  | | |  |
| Alpha Linoleic Acid Metabolism | | | | | | | | | | | | | | | | | | |  |
| Si037628 | Jasmonate ZIM Domain-Containing Protein | 0 | 2.05 | | | UP | | | - | | | | - | | | - | | |  |
|  |  |  |  | | | |  | |  | | | | |  | | | |  |  |
|  |  |  |  | | | |  | |  | | | | |  | | | |  |  |
| MAPK Signalling Pathways | | | | | | | | | | | | | | | | | | |  |
| Phytoharmones |  |  | |  | | | |  |  | | | | |  | | | |  |  |
| Si016376 | Ethylene Receptor | 0 | | 4.43 | | | | UP | - | | | | | - | | | | - |  |
| Si036560 | Mitogen-Activated Protein Kinase Kinase 9 | 6.82E-08 | | 1.27 | | | | UP | - | | | | | - | | | | - |  |
| Si009909 | Ethylene-Insensitive Protein 3 | 2.11E-03 | | 4.92 | | | | UP | - | | | | | - | | | | - |  |
| Si037098 | Basic Endochitinase B | 4.47E-06 | | 1.57 | | | | UP | 6.19E-06 | | | | | 5.58 | | | | UP |  |
| Si002730 | Transmembrane Protein 222 | 9.15E-04 | | 1.05 | | | | UP | - | | | | | - | | | | - |  |
|  |  |  | |  | | | |  |  | | | | |  | | | |  |  |
| Salt, Drought Osmotic Stress | | | | | | | | | | | | | | | | | | |  |
| Si001708 | Protein Phosphatase 2C | 0 | | 1.89 | | | | UP | - | | | | | - | | | | - |  |
| Si036041 | Serine/Threonine-Protein Kinase SRK2 | 0 | | 2.12 | | | | UP | - | | | | | - | | | | - |  |
| Si021980 | Mitogen-Activated Protein Kinase Kinase Kinase 17/18 | 0 | | 3.09 | | | | UP | 1.08E-04 | | | | | 2.49 | | | | UP |  |
| Si016999 | Catalase | 0 | | 3.09 | | | | UP | 0.01074 | | | | | 2.22 | | | | UP |  |
